# Supplementary material for: Topology stabilized fluctuations in a magnetic nodal semimetal
Source: Nat Commun. 2023 Aug 25;14:5182. doi: 10.1038/s41467-023-40765-1 (PMC10457388; doi:10.1038/s41467-023-40765-1)
Supplement: Supplementary file 1 — Supplementary Information [file 41467_2023_40765_MOESM1_ESM.pdf]

Supplementary Information

Topology stabilized fluctuations in a magnetic nodal semimetal

Nathan C. Drucker[1,2⋆,†], Thanh Nguyen[1,3,⋆], Fei Han[1,3,∗], Phum Siriviboon[1,4,∗], Xi Luo[5,⋆], Nina Andrejevic[6], Ziming Zhu[7], Grigory Bednik[3], Quynh T. Nguyen[4], Zhantao Chen[8], Linh K. Nguyen[4], Tongtong Liu[1,4], Travis J. Williams[9], Matthew B. Stone[9], Alexander I. Kolesnikov[9], Songxue Chi[9], Jaime Fernandez-Baca[9], Christie S. Nelson,[10], Ahmet Alatas[11], Tom Hogan[12], Alexander A. Puretzky[13], Shengxi Huang[14], Yue Yu[15,†] and Mingda Li[1,3,†]

[1]*Quantum Measurement Group, MIT, Cambridge, MA 02138, USA*

[2]*John A. Paulson School of Engineering and Applied Sciences, Harvard University, Cambridge, MA 02138, USA*

[3]*Department of Nuclear Science and Engineering, MIT, Cambridge, MA 02139, USA*

[4]*Department of Physics, MIT, Cambridge, MA 02139, USA*

[5]*College of Science, University of Shanghai for Science and Technology, Shanghai 200093, China*

[6]*Argonne National Laboratory, Lemont, IL 60439, USA*

[7]*School of Physics and Electronics, Hunan Normal University, Changsha 410081, China*

[8]*SLAC National Accelerator Laboratory, Menlo Park, CA 94025, USA*

[9]*Neutron Scattering Division, Oak Ridge National Laboratory, Oak Ridge, TN 37831, USA*

[10]*National Synchrotron Light Source II, Brookhaven National Laboratory, Upton, NY 11973, USA*

[11]*Advanced Photon Source, Argonne National Laboratory, Lemont, IL 60439, USA*

[12]*Quantum Design, Inc., San Diego, CA 92121, USA*

[13]*Center for Nanophase Materials Sciences, Oak Ridge National Laboratory, Oak Ridge, TN 37831, USA*

[14]*Department of Electrical Engineering, The Pennsylvania State University, State College, PA 16802, USA*

[15]*Department of Physics and State Key Laboratory of Surface Physics, Fudan University, Shanghai 200433, China*

[∗]These authors have contributed equally to this work.

[†]Corresponding authors. mingda@mit.edu, ndrucker@g.harvard.edu, yuyue@fudan.edu.cn

Supplementary Note 1: Samples

We synthesized high-quality single crystals of CeAlGe through the Al self-flux method. A mixture of Ce powder (Strem Chemicals, 99.9%), Al beads (Sigma-Aldrich, 99.9%) and Ge powder (Beantown Chemical, 99.999%) were weighed in a molar ratio of 1:10:1 in a glovebox and placed into a crucible. The mixture-filled crucible was flame-sealed in an evacuated quartz tube and was subsequently heated up to 1100°C from room temperature at a rate of 80°C/h. Afterwards, the mixture dwelled for 20 hours and subsequently cooled to 700°C at a rate of 3°C/h. This was followed by several days of annealing at this temperature after which centrifugation was performed to remove the excess flux. The resulting products of CeAlGe single crystals approximately half-centimeter large and have a metallic luster with lattice constants $a = 4.29$ Å and $c = 14.74$ Å as measured with powder X-ray diffraction. 10 g of CeAlGe in powder form were also prepared via a solid-state reaction for the time-of-flight neutron scattering experiments. The Ce, Al and Ge powders were weighed in a 1:1:1 molar ratio and placed in a crucible which was flame-sealed in an evacuated quartz tube. The materials were calcined at 700°C. The resulting products were ground and flame-sealed in quartz tube to be annealed at 700°C for several days. An image of co-aligned CeAlGe samples ($\sim$ mm$\times$mm in lateral size) glued onto an aluminum plate for inelastic neutron scattering measurements is shown in Supplementary Figure 1.

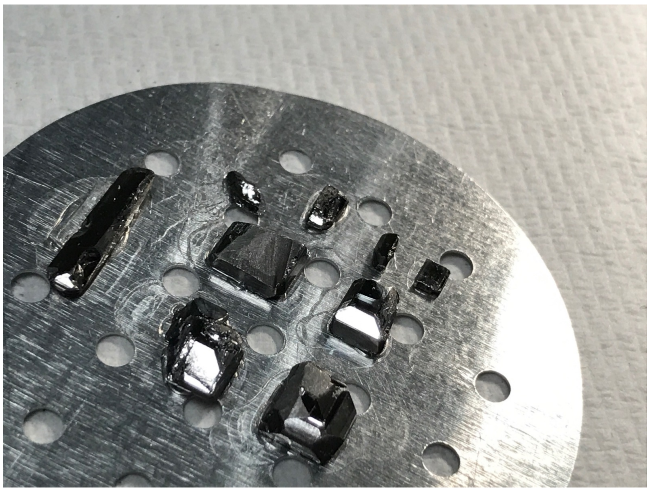

Supplementary Figure 1. Co-aligned CeAlGe single crystals. Image of co-aligned CeAlGe single crystals on an aluminum plate in preparation for inelastic neutron scattering measurements. The lateral size of each individual crystal of ~ 5 mm$\times$mm.

Supplementary Note 2: Electric transport

We perform a thinning-down process of the RAlGe (R = La, Ce) single crystals along the c-axis to a thin slab down to thicknesses of 0.265 mm (LaAlGe) and 0.370 mm (CeAlGe), as it is difficult to perform electric and thermal transport measurements on the as-grown single crystals. The contacts of the electric and thermal transport probes were made using silver epoxy H20E and copper wires.

Electrical resistance is measured for both LaAlGe and CeAlGe as a function of magnetic field using the electric transport option (ETO) of a Physical Property Measurement System (PPMS, Quantum Design) within an applied magnetic field interval of 0 T to 9 T and a temperature interval of $\sim$ 1.8 K to 300 K. These electrical resistivity measurements are performed using a standard six-probe configuration with the longitudinal and transverse resistivity probes connected to independent measurements channels. As the contacts are manually fabricated with silver epoxy, the measured data may exhibit effects of asymmetry with magnetic field due to slight misalignments of the silver contacts. Accordingly, we mitigate this effect of possible contact misalignment by averaging the longitudinal ρ_{xx} and transverse ρ_{xy} resistivities using the following equations

$$\rho_{xx}(B) = \frac{\rho(+B) + \rho(-B)}{2} \tag{1}$$

$$\rho_{xy}(B) = \frac{\rho(+B) - \rho(-B)}{2}\left(\frac{L}{W}\right) \tag{2}$$

where $\rho(+B)$, $\rho(-B)$ indicate the measured resistivity at positive and negative values of the magnetic field, respectively, while L and W designate the length and the width of the sample. These values of ρ_{xx} are used in the second derivative plots of Supplementary Figure 2a.

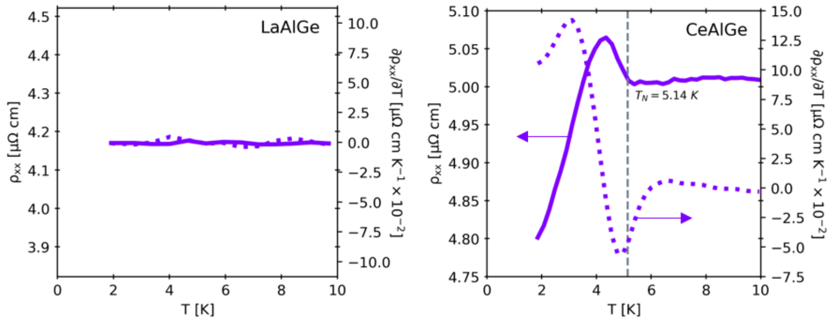

Supplementary Figure 2. Longitudinal resistivity near the transition temperature of CeAlGe. Longitudinal resistivity (solid line; left axis) shown alongside the first derivative (dotted line; right axis) of LaAlGe and CeAlGe. The y-axis range is chosen to be the same for both samples to contrast the magnetic transition in CeAlGe. The initial jump from the longitudinal resistivity is found to be T = 5.14 K through a fitting of the peak and the linear portion above the transition temperature. The center of the peak for ρ_{xx} in CeAlGe is $T \sim$ 4.3 K.

A plot showcasing the transition temperature of CeAlGe observed in ρ_{xx} is shown in Supplementary Figure 2 in comparison to data of LaAlGe. The plot of $\rho_{xx}(B)$ at different fields for CeAlGe is shown in Fig. 1b whereas that for LaAlGe can be observed in Supplementary Figure 3. The measured longitudinal resistivity ρ_{xx} is symmetric with respect to external magnetic field and does not show any signs of Shubnikov-de Haas (SdH) oscillations up to 9 T at the lowest measured temperature of $\sim$ 1.8 K. From our data, the longitudinal resistivity ρ_{xx} of LaAlGe demonstrates normal metallic behavior which is in sharp contrast to the behavior seen in CeAlGe, which is described in the main

text.

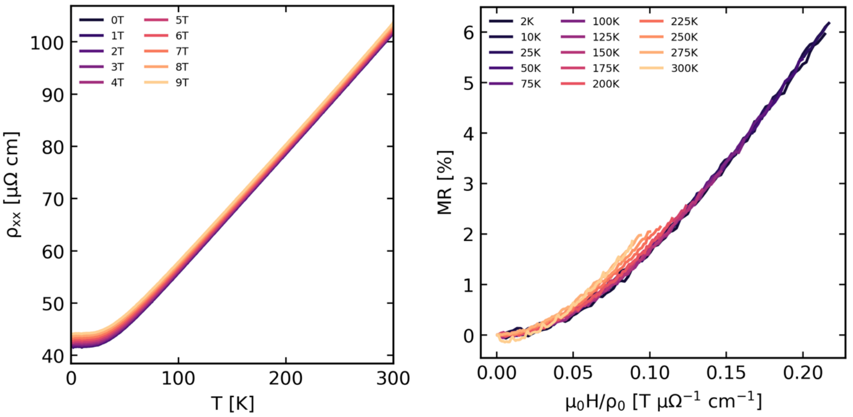

Supplementary Figure 3. Longitudinal resistivity of LaAlGe. (left) Longitudinal resistivity ρ_{xx} with respect to temperature up to 300 K from 0 T to 9 T. (right) Magnetoresistance (MR) plotted with respect to $\mu_0 H/\rho_0$ at temperatures ranging from 2 K to 300 K.

We quantify the magnetoresistance (MR) of the samples using the following definition

$$\mathrm{MR} = \frac{\rho(B) - \rho(B=0)}{\rho(B=0)} \times 100\%. \tag{3}$$

The temperature dependence and the applied magnetic field dependence of the MR for CeAlGe is plotted in the main text in Figs. 3a and 3d, respectively. The field-dependence of the MR for LaAlGe is plotted in Supplementary Figure 3 as a MR versus $\mu_0 H/\rho(B=0)$ [1] at different temperatures. The MR of LaAlGe increases quadratically with $\mu_0 H/\rho(B=0)$ which is again consistent with normal metallic behavior. LaAlGe appears to exhibits single-band behavior at low temperatures as evidenced by the universal scaling of MR at these temperatures.

Furthermore, we show data of the measured Hall (transverse) resistivity ρ_{xy}, which is antisymmetric with external magnetic field, in Supplementary Figure 4 for LaAlGe and CeAlGe. The magnitudes of the transverse resistivities ρ_{xy} of both compounds are quantitatively comparable and exhibit a linear increase in magnitude with magnetic field. For CeAlGe, there is a slight rise in the magnitude of ρ_{xy} at low temperatures with decrease in temperature.

The carrier concentrations and mobilities of LaAlGe and CeAlGe were extracted using a one-band model as Supplementary Figure 4 suggests single-band behavior for LaAlGe. The Hall coefficient for both materials is positive, thereby indicating that the samples are hole-dominated as seen in Supplementary Figure 5. The carrier concentrations of both samples are comparable, especially at low temperatures, with LaAlGe having an overall larger carrier concentration. CeAlGe has a larger hole

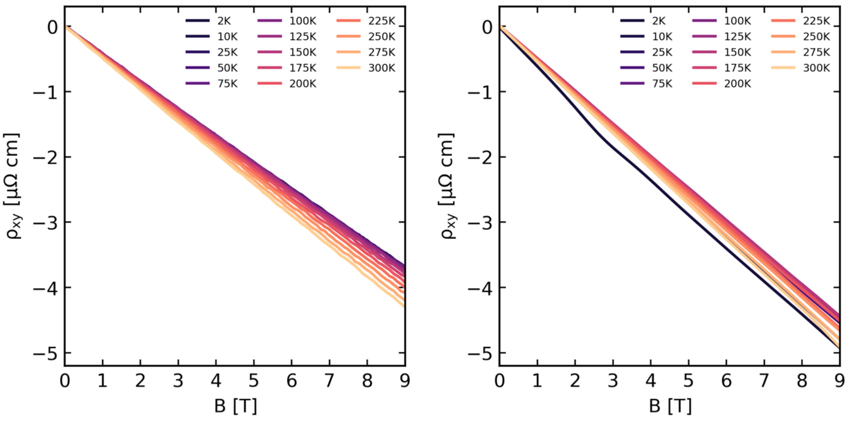

Supplementary Figure 4. Transverse resistivity of RAlGe (R = La, Ce). Transverse resistivity ρ_{xy} with respect to applied magnetic field along the c-direction of LaAlGe (left) and of CeAlGe (right) samples up to 9 T from 2 K to 300 K.

mobility at lower temperatures, but decreases below that of LaAlGe at larger temperatures.

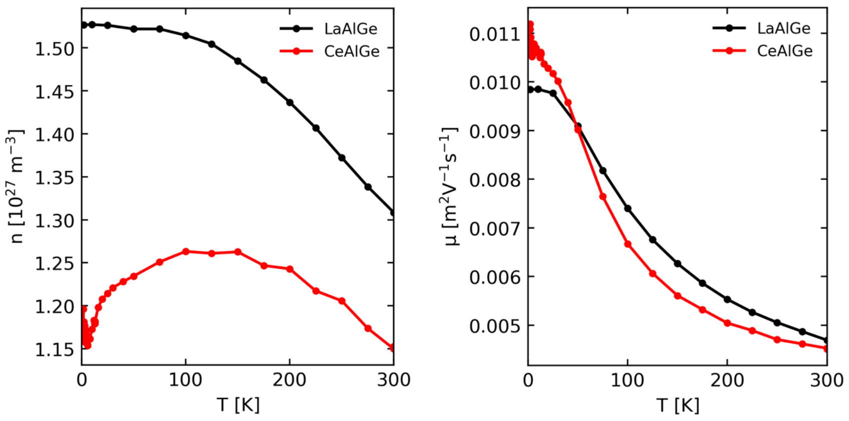

Supplementary Figure 5. Carrier densities and mobilities of RAlGe (R = La, Ce). Carrier densities (left) and mobilities (right) of LaAlGe (black) and CeAlGe (red) as a function of temperature extracted using a one-band model. The positive Hall coefficient indicates hole-dominated carriers for both.

Supplementary Note 3: Thermal transport

The thermal conductivity of the CeAlGe sample is measured with the thermal transport option (TTO) of the PPMS (Quantum Design). Similar to the electric transport measurements of Section , we perform a thinning-down process of the sample to a thickness of 0.260 mm for the purpose of facilitating the measurement. The contacts are fabricated using silver epoxy H20E.

With the device geometry shown in Supplementary Figure 6a, the ends of the thinned crystal serve as the heat source and the heat sink, with thermometers positioned along the transverse direction to record the temperature difference. The magnetic field is applied perpendicular to the device, along the c-axis of the crystal. The resulting total thermal conductivity is slightly asymmetric with respect to

positive and negative fields due to the effect of possible contact misalignment. To correct for this effect, the longitudinal and transverse components of the thermal resistivities are averaged over as follows

$$\rho_{th,xx}(B) = \frac{\kappa(+B) + \kappa(-B)}{2\kappa(+B)\kappa(-B)} \tag{4}$$

$$\rho_{th,xy}(B) = \frac{\kappa(+B) - \kappa(-B)}{2\kappa(+B)\kappa(-B)} \left(\frac{L}{W}\right), \tag{5}$$

where κ is the measured total thermal conductivity and L, W represent the length- and width-wise distance, respectively, between the two thermometers measuring the hot and cold ends of the sample. The longitudinal (κ_{xx}) and transverse (κ_{xy}) thermal conductivity are subsequently calculated using

$$\kappa_{xx} = \frac{\rho_{th,xx}}{\rho^2_{th,xx} + \rho^2_{th,xy}} \tag{6}$$

$$\kappa_{xy} = \frac{\rho_{th,xy}}{\rho^2_{th,xx} + \rho^2_{th,xy}}. \tag{7}$$

The transverse thermal conductivity κ_{xy} of CeAlGe is shown in Supplementary Figure 6b which demonstrates a linear increase in magnitude with applied magnetic field. Supplementary Figure 6c shows the longitudinal thermal conductivity κ_{xx} as function of temperature.

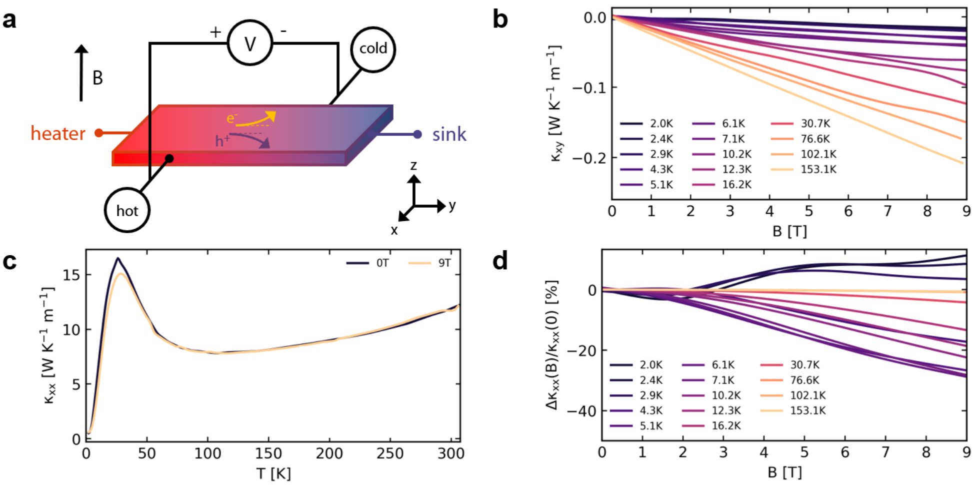

Supplementary Figure 6. Thermal transport of CeAlGe. **(a)** Device geometry for the thermal transport option (TTO) of the PPMS. Heat is injected along the longitudinal direction with thermometers placed along the transverse direction. The magnetic field is applied perpendicular to the sample in the c-direction. **(b)** Transverse thermal conductivity κ_{xy} versus magnetic field at different temperatures. **(c)** Longitudinal thermal conductivity κ_{xx} versus temperature at 0 T and 9 T. **(d)** Magneto-thermal conductivity with respect to applied magnetic field at different temperatures.

Furthermore, in analogy to the magnetoresistance (MR) defined for electric transport, we define the

magneto-thermal conductivity MC_{th} by the following equation

$$MC_{th} = \frac{\Delta\kappa_{xx}(B)}{\kappa_{xx}(B=0)} = \frac{\kappa_{xx}(B) - \kappa_{xx}(B=0)}{\kappa_{xx}(B=0)} \times 100\%. \tag{8}$$

As shown in Supplementary Figure 6d, the magneto-thermal conductivity for CeAlGe is negative above the magnetic transition, but exhibits two kinks at temperatures below the onset of ferrimagnetic order as a result of a spin alignment with the applied magnetic field. This effect is discussed further in main text as seen in Fig. 2.

Supplementary Note 4: Dilatometry

Sample dilation was measured with an ultrasensitive differential capacitive dilatometer produced by Quantum Design [2] inserted into a PPMS Dynacool. At each temperature, data was taken at both positive and negative magnetic field and the resulting dilatometry was symmetrized.

Supplementary Note 5: Phase diagrams

Transport phase diagrams are computed from data taken at different temperatures and magnetic fields that were interpolated using a triangular mesh. For each transport quantity, numerical first and second derivatives are taken with respect to temperature or magnetic field. Supplementary Figure 7 shows the resulting phase diagrams for the first derivatives with respect to the magnetic field for the electric resistivity and the thermal conductivity. The construction of the phase diagrams in Fig. 2 of the main text focuses on the second derivative in which the phase boundaries correspond to the extrema of the colormaps made using the first derivative.

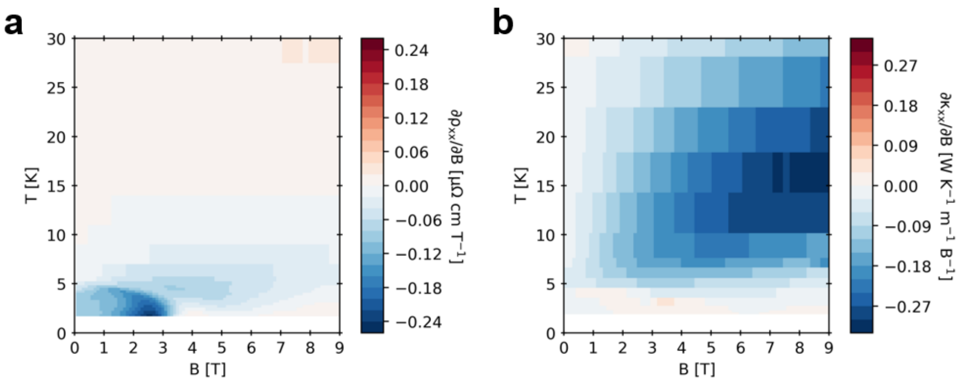

Supplementary Figure 7. Transport phase diagrams. (a) Temperature and field dependence of the first derivative of the longitudinal electrical resistivity ρ_{xx} with respect to magnetic field. **(b)** Temperature and magnetic field dependence of the first derivative of the longitudinal thermal conductivity κ_{xx} with respect to magnetic field. Red indicates positive values while blue indicates negative values.

Supplementary Note 6: Magnetization

We measure the direct current (DC) magnetization of a sample with a mass of 7 mg in a Magnetic Property Measurement System (MPMS3, Quantum Design). DC magnetization is measured from −7 T to 7 T at temperature intervals of 0.5 K from 2 K to 25 K. The phase diagrams shown in Supplementary Figure 8 are computed with a triangular interpolation scheme based on the data.

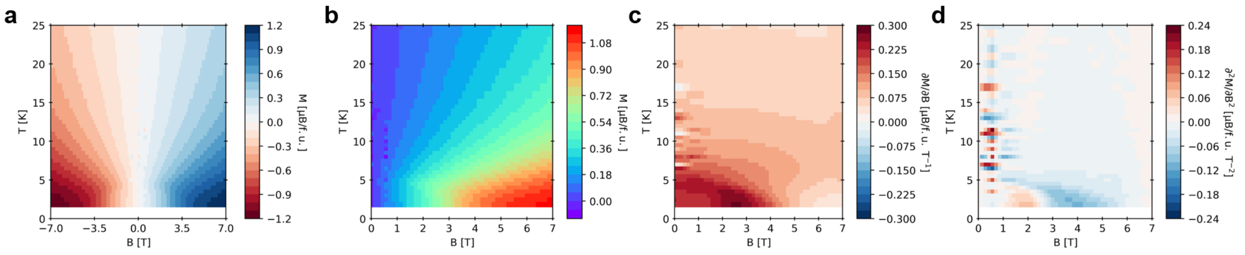

Supplementary Figure 8. DC magnetization phase diagrams. **(a)** Colormap of the low temperature magnetization dependence on applied magnetic field (along *c*-axis) and temperature. **(b)** Antisymmetrized version of **(a)**. Temperature and magnetic field dependence of the **(c)** first and **(d)** second derivatives of magnetization with respect to magnetic field.

Supplementary Note 7: Neutron diffraction

Examining the quasielastic scattering, which is integrated over an energy range of −0.5 meV to 0.5 meV reveals structural information as demonstrated in Supplementary Figure 9. In Supplementary Figure 9a, the intensity is integrated over $k = [-0.1, 0.1]$ to show the $(h, 0, l)$ peaks, which confirm the crystal structure along with powder X-ray diffraction. In Supplementary Figure 9b, when k is integrated from $k = [0.35, 0.45]$, we observe diffraction peaks with momentum transfer $\Delta\mathbf{k} = (-1.38, 0.4, \pm 2)$ corresponding to an incommensurate wave vector $\mathbf{q}_m = \mathbf{Q}_{Bragg} - \Delta\mathbf{k} = (-0.38, 0.4, 1)$ which can be related by symmetry to those found in resonant elastic X-ray diffraction.

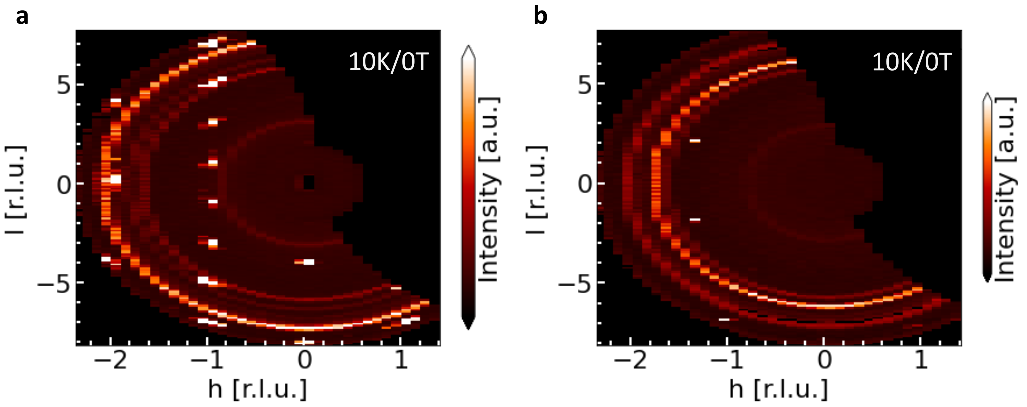

Supplementary Figure 9. Quasi-elastic neutron scattering Time of flight diffraction in the *h*-*l* plane averaged over energy transfer of $[-.5, .5]$ meV **a,** Structural Bragg peaks revealed when $k = 0$. **b,** Incommensurate peaks when k is averaged over the range of $[.35, 45]$ which match the wavevector of REXS data shown in main text.

Supplementary Note 8: Crystal field excitations

We perform time-of-flight INS measurements on single crystals of CeAlGe at different combinations of temperature (1 K, 10 K) and external magnetic field (0 T, 1 T, 8 T) using an incident neutron energy of $E_i = 240$ meV as shown in Supplementary Figure 10a to obtain direct information on the crystal electric field (CEF) excitations within this compound. The presence of CEF effects has been previously studied in other cerium intermetallics [3–5] as originating from a splitting of the six-fold $J = 5/2$ Ce^{3+} multiplet into three doublets. Indeed, six prominent CEF excitation lines can be observed in the wavevector-energy intensity plots that do not disperse with magnitude of the wavevector $|\mathbf{Q}|$ and are not drastically affected by temperature nor external magnetic field. In Supplementary Figure 10b, we show a crude fit of the neutron scattering intensity (integrated over $|\mathbf{Q}|$) using Gaussian peaks and a uniform background to extract the energies of these CEF excitations. The values of the crystal field excitations are as follows: 102.6(3) meV, 116.7(2) meV, 138.2(3) meV, 161.1(5) meV, 171.9(2) meV, and 181.1(1) meV.

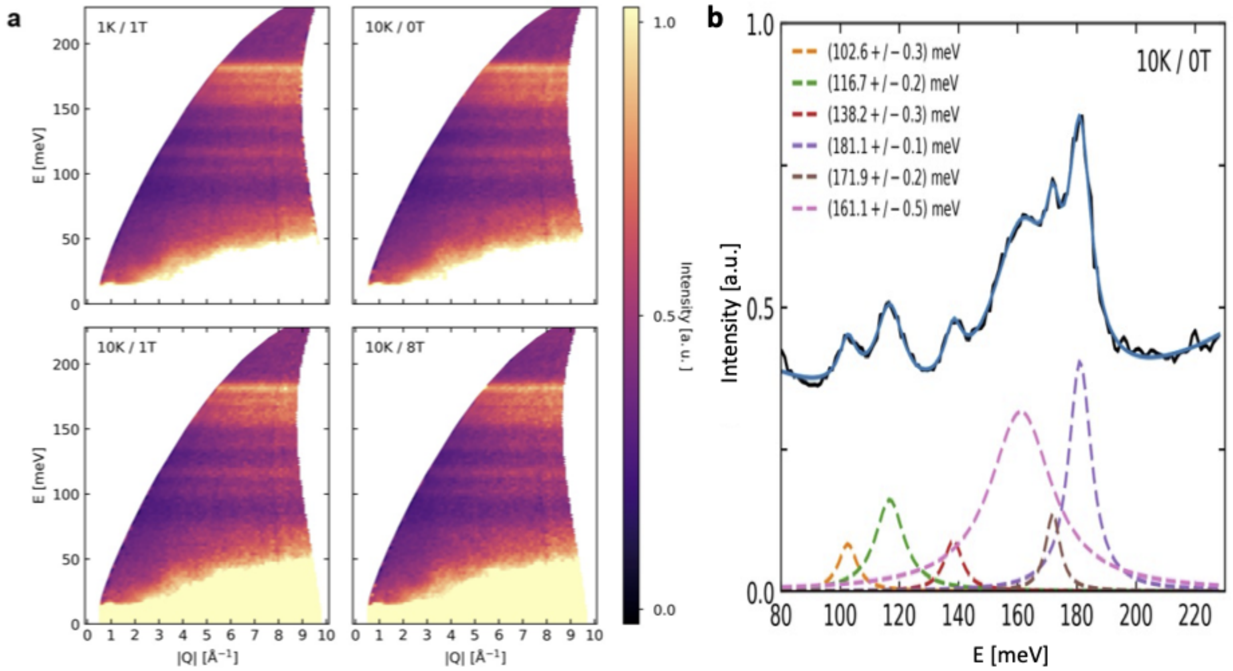

Supplementary Figure 10. Crystal field excitations in CeAlGe. (a) Powder averaged neutron intensity plots measured with an incident energy of $E_i = 240$ meV at various combinations of temperature and applied magnetic field. The horizontal and vertical axes denotes momentum $|\mathbf{Q}|$ and energy transfers E, respectively. The crystal field excitations are observed as horizontal lines (constant energy) with respect to Q between 80 meV to 170 meV, which do not change significantly with temperature or magnetic field. **(b)** The measured neutron scattering intensity integrated over $|Q|$ obtained from $E_i = 240$ meV data sets for CeAlGe samples at 10 K and 0 T. The experimental data (black line) is fitted (blue line) with a sum of Gaussian peaks with a uniform background to extract the energies of the crystal field excitations. The individual Gaussian peak contributions are plotted as dashed lines and the value of the peak center is shown in the legend.

Supplementary Note 9: Resonant elastic X-ray scattering measurements

High-precision hard X-ray scattering measurements were performed at Beamline 4-ID of the National Synchrotron Light Source II (NSLS-II) at Brookhaven National Laboratory. The sample (approximately 3.6 mm $\times$ 2 mm $\times$ 0.2 mm in size) is mounted using GE varnish onto a copper mount in a closed-cycle displex cryostat. The orientation is a vertical scattering geometry in reflection mode (Supplementary Figure 11a) with the a- and c-axes in the vertical scattering plane. To reach temperatures below the magnetic transition temperature of 4.5 K, the displex is equipped with a Joule-Thomson stage. Measurements are performed at temperatures of 4.2 K, 5 K, 7.5 K, 10 K, 12.5 K, 15 K, 20 K, 25 K, 30 K, 35 K, 40 K, 45 K, 50 K, and 300 K.

Initial measurements for crystal alignment and determination of the scattering matrix were performed using an avalanche photodiode detector. A photon energy of 6.164 keV, corresponding to the Ce L_2 edge, is selected using a cryogenically-cooled Si(111) double-crystal monochromator. The photon energy was varied between values slightly below (6.124keV) and slightly above (6.204 keV) the resonance peak to monitor the energy dependence (Supplementary Figure 12). The incident photon is polarized perpendicular (σ) to the scattering plane. Both the sigma-sigma (σ-σ') and sigma-pi (σ-π') scattering channels, corresponding to charge and magnetic scattering respectively (Supplementary Figure 11b), are measured using an LiF(220) polarization crystal analyzer with background signal suppression and a Vortex Si drift detector.

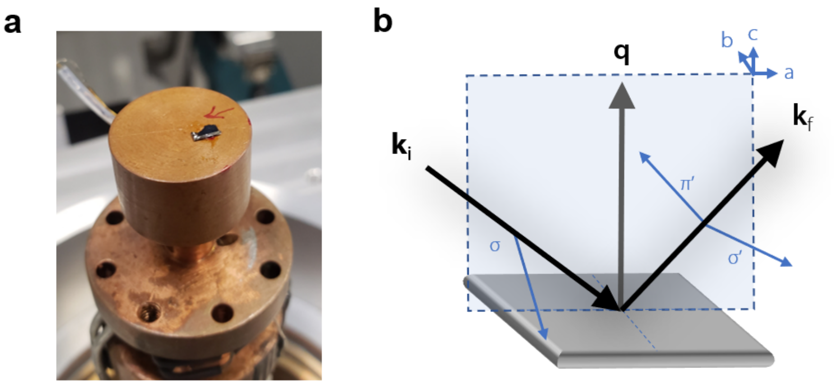

Supplementary Figure 11. Resonant elastic X-ray scattering measurements. (a) Sample of CeAlGe oriented with the a- and c-axes in the vertical scattering plane on a copper mount. The red arrow indicates the direction of the incoming photon beam. The reflective surface is in the ab-plane (a, b are equivalent) and the c-axis points out of the plane. **(b)** Schematic of the scattering plane configuration where $\mathbf{k_i}$ ($\mathbf{k_f}$) is the incoming (outgoing) wavevector, $\mathbf{q} = \mathbf{k_f} - \mathbf{k_i}$ is the momentum transfer and σ (π) is the perpendicular (parallel) polarization of the photon with reference to the scattering plane. Polarizations for outgoing photons are denoted with a prime.

REXS measurements of the nuclear Bragg peaks at (00L) where L = 4, 8 and at (10L) where L = 7,

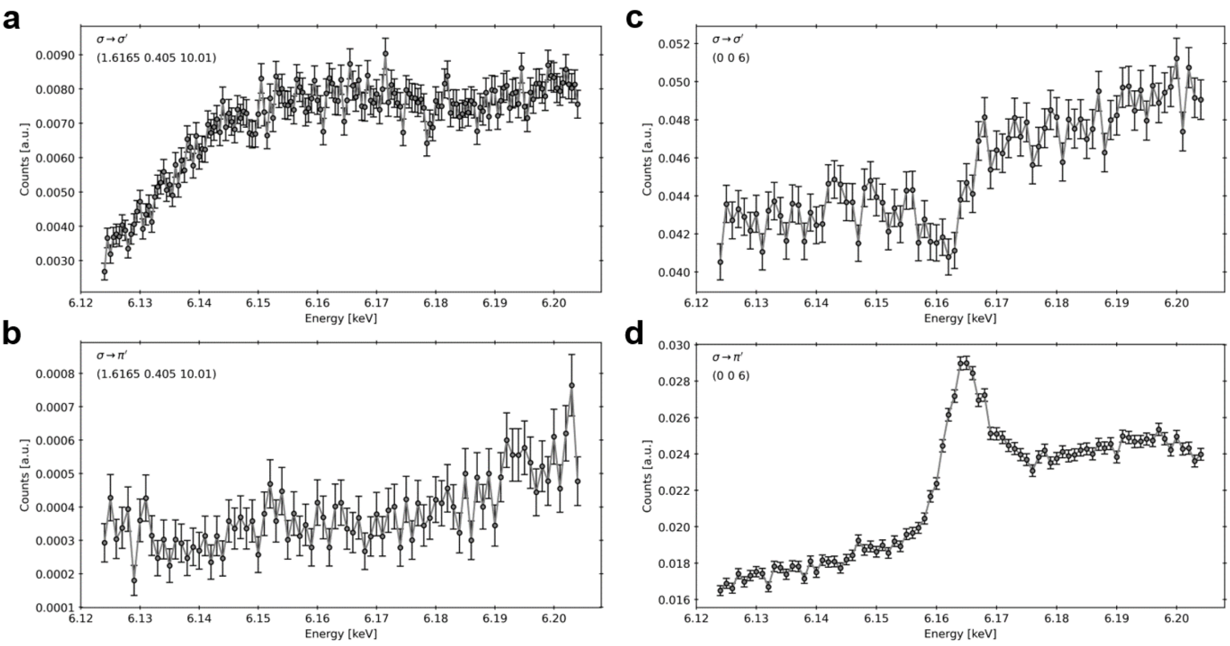

Supplementary Figure 12. Energy scans across the resonance peak of the Ce L_2 edge. Normalized intensity with respect to the monitor count plotted versus incident photon energy in the **(a)** σ-σ' and **(b)** σ-π' scattering channels at the incommensurate peak. Similar **(c)** σ-σ' and **(d)** σ-π' scattering channel measurements for the (006) peak. The energy of the resonance peak of the Ce L_2 edge is 6.164 keV.

9, 11 at different temperatures between 4 K and 50 K are shown in Supplementary Figures 13 and 14. The counts are normalized to the total number of counts at the monitor. These peaks corresponds to those expected from the crystal structure of CeAlGe. There are some secondary, extra peaks that can be identified, which is unlikely to be incommensurate peaks, but rather due to minor stacking faults in the material similar to those reported in Ref. [6]. The σ-π' scattering channel, corresponding to magnetic scattering, is a factor of 10^3 lower in intensity to that of the σ-σ' scattering channel, possibly due to intensity leakage from the analyzer not being perfectly aligned 90° away.

Supplementary Figures 15a-15c display the REXS measurements taken of the incommensurate peaks at $(1.6165, \pm 0.405, 10.01)$ along the k direction in the σ-σ' and σ-π' scattering channels. The spectra are taken at different temperatures between 4 K and 50 K and the intensity is normalized to the total number of counts of the monitor. While the intensity of the σ-π' is significantly lower than that of the σ-σ', we believe that the measured intensity of σ-π' channel here is not solely due to leakage of the σ-σ' channel due to imperfect suppression by the polarization analyzer for two reasons. 1) The ratio between the intensity of the σ-π' scattering channel to that of the σ-σ' is not the factor of 10^3 as measured for the Bragg peaks, as mentioned previously. 2) The temperature dependence between the two scattering channels is different between the channels as shown in Supplementary Figures 15d-15e. Whereas the measured peak intensity for the incommensurate peaks in σ-σ' seemingly decrease linearly with

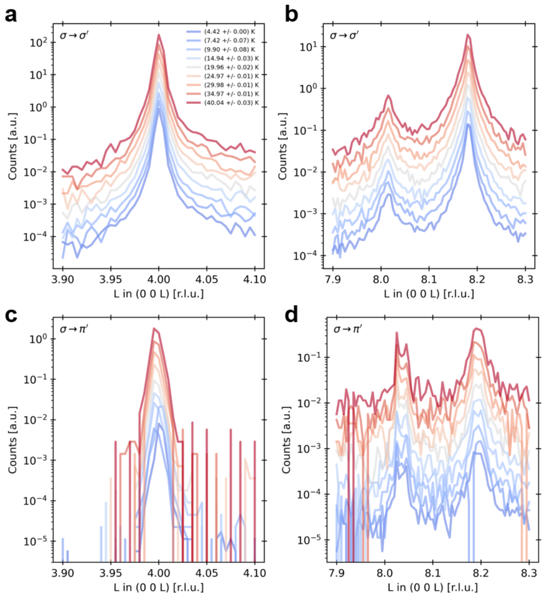

Supplementary Figure 13. REXS measurements across the (0 0 L) Bragg peaks. Normalized intensity with respect to the monitor count plotted versus L in (0 0 L) in reciprocal lattice units near **(a)** (0 0 4) and **(b)** (0 0 8) for the σ-σ' scattering channel at different temperatures. **(c-d)** Similar for the σ-π' scattering channel. The y-axis is in log scale and the spectra are sequentially shifted by a factor of 2 for visual clarity.

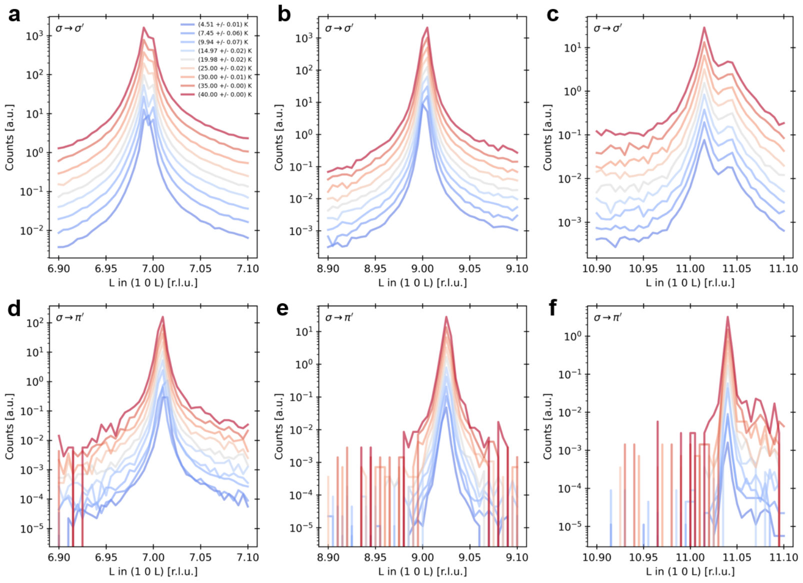

Supplementary Figure 14. REXS measurements across the (1 0 L) Bragg peaks. Normalized intensity with respect to the monitor count plotted versus L in (1 0 L) in reciprocal lattice units near **(a)** (1 0 7), **(b)** (1 0 9), and **(c)** (1 0 11) for the σ-σ' scattering channel at different temperatures. **(d-f)** Similar for the σ-π' scattering channel. The y-axis is in log scale and the spectra are sequentially shifted by a factor of 2 for visual clarity.

temperature, those for σ-π' follow a non-monotonic behavior: starting at low peak intensity, raising up to a peak value near 20 K, then decreasing with temperature. The behavior is fit to a linear regression and a third-order polynomial for the σ-σ' and σ-π' channels, respectively, for visual trends (and not based on any particular theory). The peak intensities are extracted using a Lorentzian fits of the peak with a constant background. In particular, we focus on the non-monotonic behavior of the peak intensity in the σ-π' channel in the main text.

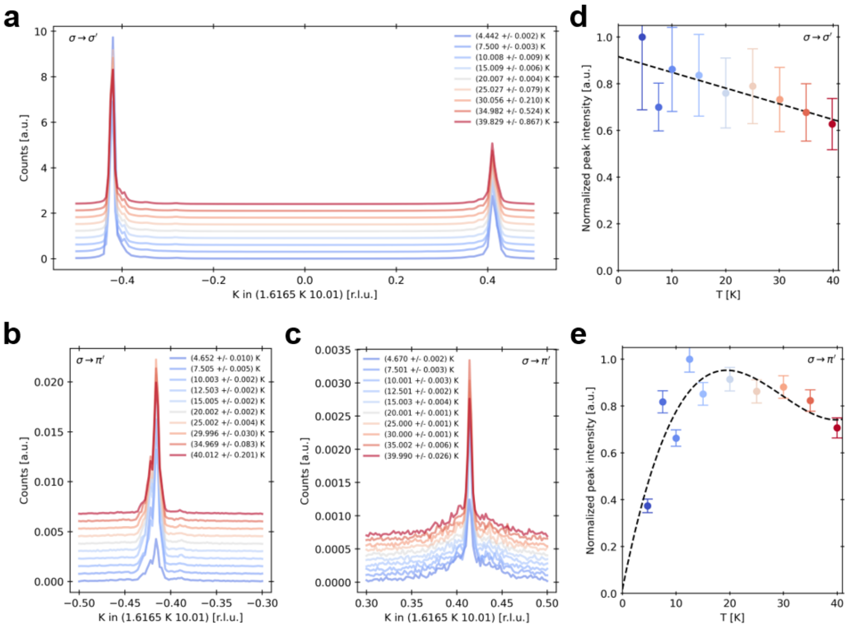

Supplementary Figure 15. REXS measurements of the incommensurate peaks. **(a)** Normalized intensity with respect to the monitor count of the two incommensurate peaks at $(1.6165, \pm 0.405, 10.01)$ for the σ-σ' scattering channel at different temperatures. **(b-c)** Similar for the σ-π' scattering channel. In subfigures **(a)**, **(b)**, and **(c)**, the spectra are shifted for visual clarity. Temperature dependence of the incommensurate peak intensity (averaged between the two peaks) for the **(d)** σ-σ' and **(e)** σ-π' scattering channels. The peak intensities are normalized to the maximum peak intensity in the temperature interval. Error bars are propagated from the Poissonian statistics of intensity counts and represent one standard deviation. The plot for the σ-σ' channel is fit to a linear regression, and that of the σ-π' channel is fit to a third-order polynomial to highlight the general trends.

Supplementary Note 10: Ab-initio calculations and Weyl node nesting

Density functional theory bandstructure calculations along Σ_0-N-Σ demonstrate the modulation of Weyl fermion bandstructures at various magnetic orderings, confirming the coupling of band topology to magnetism (Supplementary Figure 16). By examining the nesting between the Weyl nodes, based on calculations in the ferromagnetic state adapted from Ref. [7], one observes that the incommensurate magnetic wavevector lies very close to the nesting wavevector that connects the type-I W_3 Weyl nodes as shown in Supplementary Figure 17.

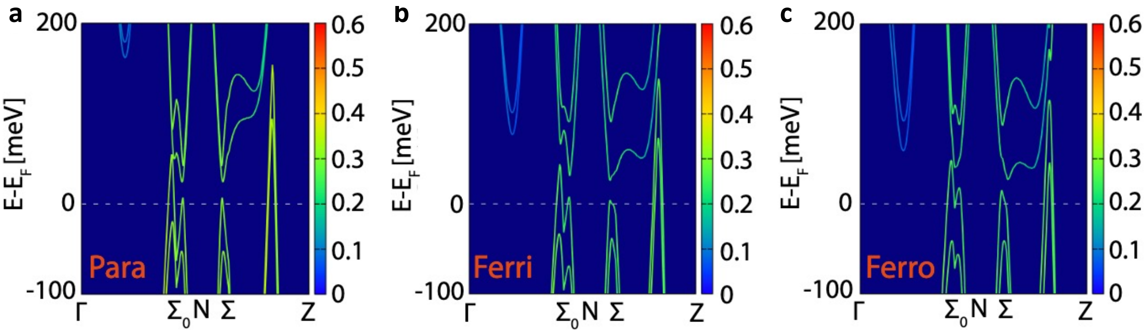

Supplementary Figure 16. Bandstructure calculations using density functional theory. Calculation of bandstructures using density functional theory for the **(a)** paramagnetic, **(b)** ferrimagnetic, and **(c)** ferromagnetic phases for CeAlGe along high-symmetry lines in the Brillouin zone. The colormap indicates the spectral intensity.

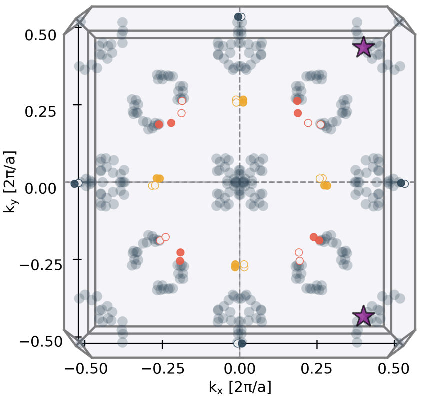

Supplementary Figure 17. Incommensurate magnetism nesting vectors. Location of Weyl nodes in the $k_z = 0$ plane within the reciprocal space unit cell adapted from Ref. [7] are indicated by colored circles. Values of the nesting vectors between the Weyl nodes calculated as the distance between them indicated by grey circles. The location of the magnetic wave vector as measured from neutron and x-ray scattering experiments is marked with a purple star, and closely matches the nesting between the W_3 Weyl nodes.

The *ab initio* calculations were computed using VASP [8–10] with projector-augmented-wave (PAW) pseudopotentials and Perdew-Burke-Ernzerhof (PBE) for the exchange-correlation energy functional [11]. This was optimized with a 6x6x2 Monkhorst-Pack grid of k-point samplings. Second- and third-order force constants were calculated using a real space supercell approach with a 3x3x1 supercell and the PHONOPY package [12] was using to obtain the second-order force constants.

Supplementary Note 11: Raman scattering

Polarization angle-resolved Raman scattering measurements were performed at the Center for Nanophase Materials Sciences (CNMS) at Oak Ridge National Laboratory (ORNL) on single crystal samples of CeAlGe and LeAlGe. These measurements are performed at $T = 300$ K. From the symmetry of the polarized Raman dependence, different phonon modes are identified in CeAlGe and LaAlGe, as plotted in Supplementary Figures 18-19, respectively, as waterfall and polar plots. Notably, several phonon modes exhibits a fourfold rotation symmetry as a function of the polarization angle.

Supplementary Note 12: Derivation of free energy correction from Kondo interaction

We began with the Hamiltonian

$$H_0 = \sum_{i,\chi,\mathbf{k}} (\chi \nu_i v_F |\mathbf{k} - \mathbf{b}_i| + b_{0,i} - \mu) \psi^\dagger_{i,\chi}(\mathbf{k}) \psi_{i,\chi}(\mathbf{k}) \tag{9}$$

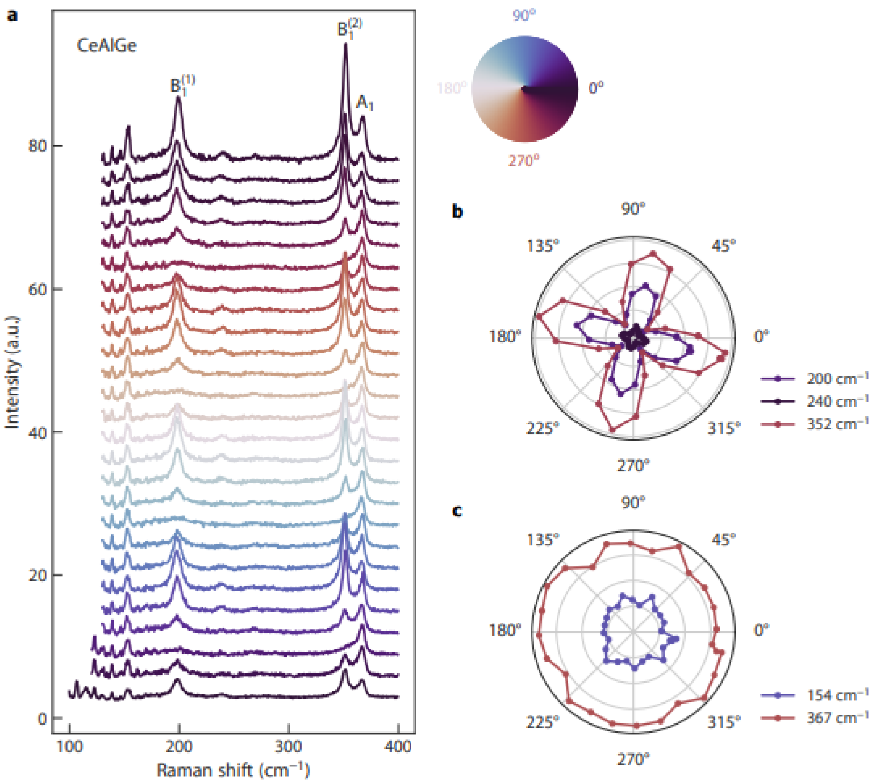

Supplementary Figure 18. Polarized Raman spectroscopy of CeAlGe. (a) Waterfall plot of polarized Raman spectra measured using a 532 nm laser excitation over a range of polarization angles. The spectra have been offset vertically for clarity. Two identified angle-dependent B_1 modes at 200 cm^{-1} and 352 cm^{-1}, and one angle-independent A_1 mode at 367 cm^{-1}, are labeled. **(b)** Polar plot of three selected modes whose intensity exhibits fourfold rotational symmetry as a function of polarization angle. Two are identified as B_1 modes, while the third mode with lower intensity is observed at 240 cm^{-1}. **(c)** Polar plot of two selected modes with conserved intensity as a function polarization angle. One is identified as an A_1 mode, while the other mode with lower intensity is observed at 154 cm^{-1}.

where the wavefunction $\psi_\nu(\mathbf{k})$ can be written in spin-z basis as

$$H_0 = \sum_{i,\chi\mathbf{k}} \chi\nu_i \begin{pmatrix} \psi^\dagger_{\nu_i\uparrow} & \psi^\dagger_{\nu_i\downarrow} \end{pmatrix} v_F \begin{pmatrix} k^i_z & k^i_z - ik^i_y \\ k^i_z + ik^i_y & -k^i_z \end{pmatrix} \begin{pmatrix} \psi_{\nu_i\uparrow} \\ \psi_{\nu_i\downarrow} \end{pmatrix} + (b_{0,i} - \mu)(\psi^\dagger_{\nu\uparrow}\psi_{\nu_i\uparrow} + \psi^\dagger_{\nu_i\downarrow}\psi_{\nu_i\downarrow}) \quad (10)$$

where $\mathbf{k}^i = \mathbf{k} - \nu\mathbf{b}_i$. To diagonalize, we can choose the basis where the spin is aligned and anti-align with the momentum $\mathbf{k}^i = k(\sin\theta^i\cos\phi^i, \sin\theta^i\sin\phi^i, \cos\theta^i)$ as followed.

$$\begin{pmatrix} \psi^\dagger_{\nu+} & \psi^\dagger_{\nu-} \end{pmatrix} = \begin{pmatrix} \psi^\dagger_{\nu\uparrow} & \psi^\dagger_{\nu\downarrow} \end{pmatrix} \begin{pmatrix} \cos(\theta^i/2) & -e^{-i\phi^i}\sin(\theta^i/2) \\ e^{i\phi^i}\sin(\theta^i/2) & \cos(\theta^i/2) \end{pmatrix} \quad (11)$$

In this form, we can easily write the finite temperature Green function of the electron as

$$G^{\nu,\chi}_{i,k} = G^{\nu,\chi}(\omega_n, \mathbf{k}) = (-i\omega_n + \epsilon_{i,\nu,\chi}(\mathbf{k}))^{-1} \quad (12)$$

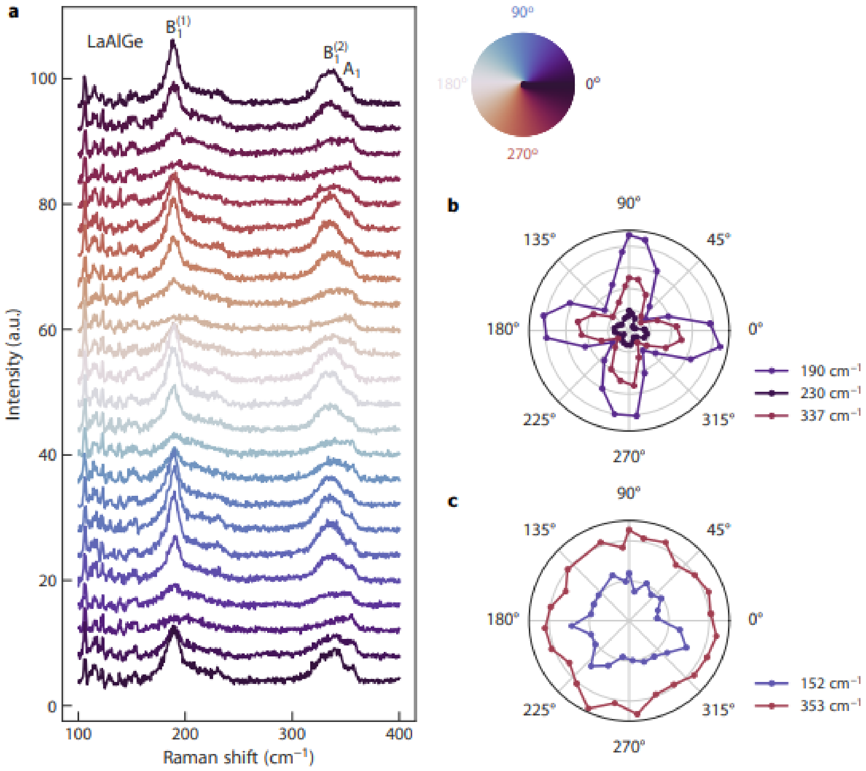

Supplementary Figure 19. Polarized Raman spectroscopy of LaAlGe. (a) Waterfall plot of polarized Raman spectra measured using a 532 nm laser excitation over a range of polarization angles. The spectra have been offset vertically for clarity. Two identified angle-dependent B_1 modes at 190 cm^{-1} and 337 cm^{-1}, and one angle-independent A_1 mode at 353 cm^{-1}, are labeled. **(b)** Polar plot of three selected modes whose intensity exhibits fourfold rotational symmetry as a function of polarization angle. Two are identified as B_1 modes, while the third mode with lower intensity is observed at 230 cm^{-1}. **(c)** Polar plot of two selected modes with conserved intensity as a function polarization angle. One is identified as an A_1 mode, while the other mode with lower intensity is observed at 152 cm^{-1}.

with $\epsilon_{i,\nu,\chi}(\mathbf{k}) = \nu\chi v_F|\mathbf{k}^i| + (b_{0,i} - \mu)$. Then, we assume the form of the magnetic texture as incommensurate spin wave $\mathbf{M} = M(\cos \mathbf{q}_m \cdot r, \sin \mathbf{q}_m \cdot r, 0)$ and rewrite the wave function in non-interacting basis (Supplementary Equation 11), we can rewrite interaction Hamiltonian as

$$H_{int} = \sum_{\mathbf{k},\nu,\alpha,\beta} \frac{K_\nu M}{2} \left(\psi^\dagger_{\nu\alpha}(\mathbf{k}) V_{\alpha\beta} \psi_{\nu,\beta}(\mathbf{k}+\mathbf{q}) + h.c.\right) \tag{13}$$

with

$$V_{++} = e^{i\phi} \cos\left(\theta'/2\right) \sin(\theta/2) \tag{14}$$

$$V_{--} = e^{i\phi'} \cos\left(\theta'/2\right) \sin(\theta/2) \tag{15}$$

$$V_{+-} = \cos(\theta/2) \cos\left(\theta'/2\right) \tag{16}$$

$$V_{-+} = -e^{i\phi} e^{i\phi'} \sin(\theta/2) \sin\left(\theta'/2\right) \tag{17}$$

which corresponds to the action

$$S_{int}[\psi^{\dagger},\psi]=\sum_{k,\nu}\frac{K_{\nu}M}{2}\sum_{\alpha\beta}\Big(\psi^{\dagger}_{\nu\alpha}(k)V_{\alpha\beta}\psi_{\nu,\beta}(k+q)+h.c.\Big) \tag{18}$$

with $k=(i\omega_n,\mathbf{k})$ and $q=(0,\mathbf{q})$ The nth-order correction to the free energy can be written as

$$F^{(n)}=-T\frac{(-1)^n}{n!}\langle S_{int}[\psi^{\dagger},\psi]^n\rangle_0^c \tag{19}$$

where $\langle ...\rangle_0^c$ corresponds to the link-cluster diagram. Due to the nature of the interaction, we can see that only the even-ordered term survives. The second-order contribution can then be calculated as

$$F^{(2)}=-T\sum_{\nu}\left(\frac{K_{\nu}M}{2}\right)^2\sum_{k,\alpha,\beta}-G_{\alpha}(k)G_{\beta}(k+q)V_{\alpha\beta}\bar{V}_{\beta\alpha} \tag{20}$$

By summing over Matsubara frequency, one yield

$$F^{(2)}=\sum_{\nu}\left(\frac{K_{\nu}M}{2}\right)^2\sum_{\mathbf{k},\alpha,\beta}-\frac{n_{\nu\beta}(\mathbf{k}+\mathbf{q}_m)-n_{\nu\alpha}(\mathbf{k})}{\epsilon_{\nu\beta}(\mathbf{k}+\mathbf{q}_m)-\epsilon_{\nu\alpha}(\mathbf{k})}V_{\alpha\beta}\bar{V}_{\beta\alpha} \tag{21}$$

Consider a contribution from a pair of WPs with identical chiral charge and renormalized the momentum with the fermi energy, we can express the free energy contribution as

$$\begin{aligned}F^{(2)}=\left(\frac{KM}{2}\right)^2\sum_{\tilde{\mathbf{k}}}\Bigg[&Re(V_{++}\bar{V}_{++})\frac{n(|\tilde{\mathbf{k}}|)+n(-|\tilde{\mathbf{k}}+\mathbf{q}|))-n(-|\tilde{\mathbf{k}}|)-n(|\tilde{\mathbf{k}}+\mathbf{q}|)}{|\tilde{\mathbf{k}}+\mathbf{q}|-|\tilde{\mathbf{k}}|}\\ &-Re(V_{+-}\bar{V}_{-+})\ \frac{n(-|\tilde{\mathbf{k}}|)+n(-|\tilde{\mathbf{k}}+\mathbf{q}|))-n(|\tilde{\mathbf{k}}|)-n(|\tilde{\mathbf{k}}+\mathbf{q}|)}{|\tilde{\mathbf{k}}+\mathbf{q}|+|\tilde{\mathbf{k}}|}\Bigg]\end{aligned} \tag{22}$$

Where $Re(V_{++}\bar{V}_{++})=Re(V_{--}\bar{V}_{--})$ and $Re(V_{+-}\bar{V}_{-+})=Re(V_{-+}\bar{V}_{+-})$. The first term corresponds to the intra-band scattering, while the second originates from the inter-band scattering. This expression depends on three energy scales: μ, $|\mathbf{q}|$, and T. For $\mu=0$, we can express the free energy correction as the function of angular dependence $\mathbf{q}/|\mathbf{q}|$, the energy scale of $|\mathbf{q}|$, and temperature scale T.

$$F^{(2)}=\left(\frac{KM}{2}\right)^2\left[f_1\left(\frac{\mathbf{q}}{|\mathbf{q}|},|\mathbf{q}|,T\right)-f_2\left(\frac{\mathbf{q}}{|\mathbf{q}|},|\mathbf{q}|,T\right)\right] \tag{23}$$

Supplementary References

1. Ziman, J. M. *Electrons and Phonons: the Theory of Transport Phenomena in Solids* (Clarendon Press; Oxford University Press, 2001).

2. Martien, D. *et al.* An Ultrasensitive Differential Capacitive Dilatometer. *IEEE Trans. Magn.* **55,** 1–4 (2019).

3. Adroja, D. T., Rainford, B. D. & Neville, A. J. Crystal fields and spin dynamics of hexagonal CeTSn compounds (T = Cu, Ag and Au). *J. Phys. Condens. Matter* **9,** L391–L395 (1997).

4. Oner, Y., Kamer, O., Ross, J. H., Lue, C. S. & Kuo, Y. K. Magnetic phase transitions in intermetallic CeCuGe compound. *Solid State Commun.* **136,** 533–537 (2005).

5. Sondezi-Mhlungu, B. M., Adroja, D. T., Strydom, A. M., Kockelmann, W. & Goremychkin, E. A. Inelastic neutron scattering and specific heat of CeCuGe. *J. Phys. Conf. Ser.* **200,** 012190 (2010).

6. Li, H. *et al.* Observation of Unconventional Charge Density Wave without Acoustic Phonon Anomaly in Kagome Superconductors AV_3Sb_5 (A = Rb, Cs). *Phys. Rev. X* **11,** 031050 (2021).

7. Chang, G. *et al.* Magnetic and noncentrosymmetric Weyl fermion semimetals in the RAlGe family of compounds (R = rare earth). *Phys. Rev. B* **97,** 041104(R) (2018).

8. Kresse, G. & Furthmüller, J. Efficiency of ab-initio total energy calculations for metals and semiconductors using a plane-wave basis set. *Comput. Mater. Sci.* **6,** 15–50 (1996).

9. Kresse, G., Furthmüller, J. & Hafner, J. Ab initio Force Constant Approach to Phonon Dispersion Relations of Diamond and Graphite. *EPL* **32,** 729–734 (1995).

10. Kresse, G. & Joubert, D. From ultrasoft pseudopotentials to the projector augmented-wave method. *Phys. Rev. B* **59,** 1758–1775 (1999).

11. Perdew, J. P., Burke, K. & Ernzerhof, M. Generalized Gradient Approximation Made Simple. *Phys. Rev. Lett.* **77,** 3865–3868 (1996).

12. Togo, A. & Tanaka, I. First principles phonon calculations in materials science. *Scr. Mater.* **108,** 1–5 (2015).
